# Application of Situational Leadership to the National Voluntary Public Health Accreditation Process

**DOI:** 10.3389/fpubh.2013.00026

**Published:** 2013-08-12

**Authors:** Kristina Rabarison, Richard C. Ingram, James W. Holsinger

**Affiliations:** ^1^College of Public Health, University of Kentucky, Lexington, KY, USA

**Keywords:** situational leadership, public health accreditation, accreditation, leadership, student training

## Abstract

Successful navigation through the accreditation process developed by the Public Health Accreditation Board (PHAB) requires strong and effective leadership. Situational leadership, a contingency theory of leadership, frequently taught in the public health classroom, has utility for leading a public health agency through this process. As a public health agency pursues accreditation, staff members progress from being uncertain and unfamiliar with the process to being knowledgeable and confident in their ability to fulfill the accreditation requirements. Situational leadership provides a framework that allows leaders to match their leadership styles to the needs of agency personnel. In this paper, the application of situational leadership to accreditation is demonstrated by tracking the process at a progressive Kentucky county public health agency that served as a PHAB beta test site.

## Introduction

The mission of public health, as identified by the 1988 Institute of Medicine (IOM) report, *The Future of Public Health*, is “assuring conditions in which people can be healthy” (1). A strong infrastructure is central to the mission of public health, since it supports the delivery of key public health services. The critical role infrastructure plays in assuring public health is underscored in a 2003 IOM follow up report that identified strengthening governmental public health institutions as an essential area of action for the twenty-first century. The 2003 report highlighted the key role that leadership plays in maintaining a strong public health system through the development of a competent public health workforce. It also identified the importance of leadership in such specific recommendations as making “leadership training, support, and development” a high priority for all governmental public health agencies, schools of public health, and the other entities within the public health system (2).

Successful leadership is contingent upon developing a clear mission and executing a vision to guide progress (3). Various frameworks have been developed to guide public health leaders in developing a mission and vision, including the three Core Functions of Public Health and the 10 Essential Public Health Services (EPHS) (4). While these frameworks are useful, they are macro-contextual, and may be disconnected from the day to day operations of a public health agency. The accreditation standards and measures developed by The Public Health Accreditation Board (PHAB) provide specific benchmarks to be utilized by agencies as a framework to guide their activities. While PHAB’s standards and measures can be used to guide organizational leadership, the changes associated with accreditation require strong leadership and an immediate short-term strategic plan and long-term vision based on effectiveness, efficiency, and sustainability.

Academic public health programs, as part of their curricula, educate students in leadership theories and models, and often include skill training at both the masters and doctoral levels. Students of public health rarely are provided the opportunity to practice the leadership skills developed in the classroom or to test leadership theories in real world situations prior to degree completion. This article discusses one opportunity to transfer leadership theory and practice from the classroom to the practice setting. In this instance, practice based field experience provided a public health doctoral student the opportunity to utilize concepts learned in the classroom in a practice setting, and develop a case study, based on initial and follow up interviews with public health agency personnel, focused on leadership in the context of preparing for participation in a Beta Test of the PHAB pilot standards and measures.

## Situational Leadership

Situational leadership theory suggests that leaders should adapt their leadership styles based on the readiness, current skills, and developmental level of team members (5). It provides the leader with the flexibility to assess the situation and adopt a leadership style that best fits the needs of the follower. It is particularly well suited to leading public health agencies through the accreditation process as will be demonstrated.

Utilizing Situational Leadership requires leaders to be aware of the perceptions of their followers. What leaders say they do is one thing; what followers say they want and how well their leaders meet their expectations is another (6). Given the novelty of accreditation, and the potential anxiety engendered during the different phases of the process, public health leaders need to be aware of and adapt their leadership styles to match the readiness, current skills, and developmental status of the team members engaged in accreditation, allowing the agency to successfully navigate this intricate process.

**Figure 1:**
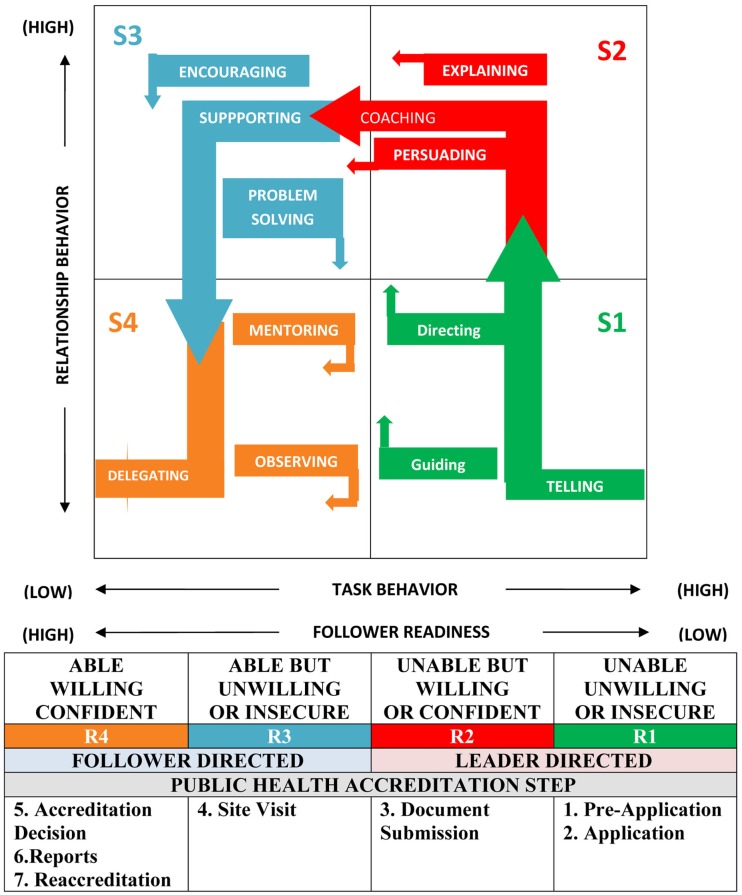
**Situational leadership and public health accreditation**. Adapted from Ref. (5).

Situational leadership is based on two behavioral categories: task behavior and relational behavior. Task behavior is “the extent to which the leader engages in spelling out the duties and responsibilities of an individual or group” (7). Relational behavior is “the extent to which the leader engages in two-way or multi-way communication if there is more than one person” (7). Thus, situational leadership provides a balance between (1) guidance and direction (task behavior), (2) socio-emotional support (relational behavior), and (3) the readiness level followers exhibit for a specific task (5). The leadership styles of situational leadership include:

1. Style 1 (S1) “Directing” characterized by “high task and low relationship” behaviors;
2. Style 2 (S2) “Coaching” characterized by “high task and high relationship” behaviors;
3. Style 3 (S3) “Participating” characterized by “high relationship and low task” behaviors;
4. Style 4 (S4) “Delegating” characterized by “low relationship and low task” behavior (5) (see Figure 1).

In situational leadership, readiness is defined as “the extent to which a follower demonstrates the ability and willingness to accomplish a specific task” (5). The major components of readiness are ability defined as “the knowledge, experience, and skill that an individual or a group brings to a particular task or activity,” and willingness is defined as “the extent to which an individual or a group has the confidence, commitment, and motivation to accomplish a specific task” (5). As seen in Figure 1, follower readiness is a continuum from low to high as followers develop ability and willingness. Leaders match their leadership style to the readiness level of their followers as follows:

1. Level 1 (R1) occurs when the follower is “unable and unwilling” to perform the task and lacks confidence, motivation, and commitment;
2. Level 2 (R2) occurs when the follower is “unable but willing” to perform the task and requires some guidance;
3. Level 3 (R3) occurs when the follower is “able but unwilling” to complete the task, possibly because of insecurity; and
4. Level 4 (R4) occurs when the follower is “willing and able” to accomplish the task with confidence (5) (see Figure 1).

## Situational Leadership and Public Health Accreditation: A Local Health Agency Case Study

While accreditation is not a new concept in the American health sector [initiatives such as The Joint Commission on Accreditation of Healthcare Organizations (JCAHO) have been a part of the health care system for decades], it is a new phenomenon in public health practice in the United States. Informal discussions concerning the accreditation of public health agencies have occurred for some time; however, accreditation received a significant boost from *The Future of the Public’s Health in the Twenty-First Century*, which stated that “despite the controversies concerning accreditation, greater accountability is needed on the part of state and local health agencies with regard to the performance of the core public health functions of assessment, assurance, and policy development and the EPHS” (8). This report led to the creation of the Exploring Accreditation project in 2004, the creation of PHAB in 2007, and ultimately the release of PHAB’s standards and measures for voluntary national accreditation in 2011.

Accreditation is a useful tool for improving the quality of services provided to the public by setting standards and evaluating performance against those standards, and has been shown to be associated with higher performing health systems. In a working paper for the Robert Wood Johnson Foundation (RWJF), Mays demonstrated that clinical quality measures for care of myocardial infarctions were lower and mortality rates higher in hospitals not participating in JCAHO accreditation when compared to JCAHO accredited healthcare facilities (9). It may be postulated that accreditation of public health agencies will have a similar effect. PHAB states that its program is intended to develop and maintain “a high-performing governmental public health system that will make us the healthiest nation.” Thus, PHAB “is dedicated to promote, improve, and protect the health of the public by advancing the quality and performance of state, local, tribal, and territorial public health departments in the United States” (10).

The PHAB accreditation process has seven steps; Pre-application, Application, Documentation Selection and Submission, Site Visit, Accreditation Decision, Reports, and Reaccreditation; and was developed after extensive review and revision, including a beta test of the process, which included 30 state, tribal, and local public health agencies (10, 11). Following an interview with the director of a local public health agency regarding the agency’s experience as a beta test site, the authors noted that the agency’s accreditation experience closely matched the four situational leadership styles in relationship to the stages of follower readiness displayed in Figure 1. As a result, a follow up interview was completed to confirm these findings, and to further discuss the application of situational leadership to the accreditation process.

The agency was well prepared for accreditation given its previous commitment to continuous quality improvement, as evidenced by its application to be a beta test site. In addition, the agency director was a member of the Kentucky Department of Public Health Quality Improvement Team prior to accepting her current position (12). This agency is also committed to performance measurement and management, having completed in 2008 a local public health system performance assessment that demonstrated a relatively high (69%) score in the overall performance of the EPHS (12).

During the initial interview with the agency director, it was apparent that leadership was viewed as a key element to accreditation success. Fostering complete organizational commitment to the process was of particular importance, including high commitment from contract and part time employes, as well as members of the local board of health.

Early in the accreditation process, particularly during the pre-application and application stages, and partially during document submission, the agency staff was relatively unfamiliar with the accreditation process (R1 follower readiness level as depicted in Figure 1), necessitating that the agency director engage in leader directed activities, primarily those shown in the S1 area in Figure 1. Such actions involved informing the agency staff of the requirements and processes of accreditation and directing them through the process with high task behaviors answering the question: what is public health accreditation? She utilized a directing style of leadership dealing with questions such as who, what, when, where, and how.

As agency staff members developed an understanding of the value of accreditation and gained some confidence through identifying their roles in the process and the documents necessary for review, they transitioned to an R2 stage of follower readiness as depicted in Figure 1, resulting in the director continuing highly directive behavior while adding high relationship behavior as well. A coaching, persuading, and/or explaining leadership style (S2 quadrant of the diagram) became important. While the leadership style was still high task, moving from direction to explanation occurred in order to answer the question, “Why is accreditation important to our agency?”.

By the time the agency was ready for document submission its personnel had sufficient confidence to transition fully to the R2 stage of readiness. There were still gaps in knowledge and ability related to the accreditation process, thus necessitating a continuation of the S2 leadership style, including coaching, explaining, and continuously persuading public health agency staff members of the value of accreditation and the importance of each individual’s role in the agency’s effort.

By the time the agency reached the PHAB’s beta test site visit phase, it had reached an R3 stage of readiness as depicted in Figure 1. As a result, leadership style was based on high relationship, low task behaviors characterized by quadrant S3. These follower-directed behaviors revolved primarily around encouraging and championing the efforts of a highly participatory agency staff, with agency leaders assuming the role of problem solvers instead of being more highly task oriented.

By the conclusion of the PHAB beta test experience, when mock accreditation feedback was provided, the agency staff members had developed to an R4 stage of readiness. The agency staff was able, willing, and confident with respect to accreditation. As a result, the leader’s style had shifted to a low task and low relational behavior approach as described by quadrant S4. The director successfully delegated the accreditation coordination task to an accreditation coordinator, thus serving as an engaged mentor.

The PHAB beta test experience allowed the agency to further develop its quality improvement, performance measurement, and management infrastructure. The agency had successfully completed the three prerequisites of PHAB accreditation by developing a community health assessment, a community health improvement plan, and a refined strategic plan with clear mission and vision statements that were ready to be adopted. In addition, a 12 member accreditation team had been formed, being led by the full time accreditation coordinator.

As a result of the commitment and intense preparation exhibited by the staff, on February 28, 2013, the agency was awarded 5-year accreditation status by PHAB.^1^ Accreditation of the agency was a direct result of the leadership exhibited by the agency’s senior leadership. The accreditation result was based on the development of a high-performing team founded on full collaboration between staff members and leaders. The use of a situational leadership approach contributed to team development. Conflict resolution was more readily accomplished by the leaders’ understanding of the needs of the staff members and the leaders’ ability to utilize an appropriate leadership style to meet the staff members’ needs. Due to the nature of the PHAB accrediting process, no ethical issues were raised by staff members during the beta test experience.

## Summary

Situational leadership theory and skills learned in the classroom were effective in understanding the leadership required to effectively guide a public health agency through the process of preparing for PHAB accreditation. This theory of leadership is an appropriate approach for leading the accreditation process due to its flexibility as a follower driven model of leadership. Given the novelty and the complexity of the accreditation process, a highly functioning team is required and situational leadership provides a framework for public health agency leaders to successfully guide their teams through the process. Use of situational leadership will ensure that public health agencies successfully develop an ongoing quality improvement and performance standards plan throughout the accreditation process. Thus, a classroom leadership theory was found to be useful as an approach to being faithful to public health’s mission to “assure conditions in which people can be healthy” (1).

## Conflict of Interest Statement

The authors declare that the research was conducted in the absence of any commercial or financial relationships that could be construed as a potential conflict of interest.

## Footnotes

^1^http://www.phaboard.org/news-room/accredited-health-departments
